# Case Report: Takotsubo Cardiomyopathy After Traumatic Brain Injury

**DOI:** 10.3389/fneur.2021.727754

**Published:** 2021-09-15

**Authors:** Fajun Wang, Joseph Darby

**Affiliations:** Department of Critical Care Medicine, University of Pittsburgh Medical Center, Pittsburgh, PA, United States

**Keywords:** case report, Takotsubo cardiomyopathy, traumatic brain injury, stress cardiomyopathy, TBI, diagnosis

## Abstract

**Introduction:** Takotsubo cardiomyopathy (TCM) or “stress cardiomyopathy” is an uncommon condition characterized by transient cardiac dysfunction with left ventricular apical ballooning in an appropriate clinical context. TCM has been observed in a variety of acute neurological conditions most prominently in patients with aneurysmal subarachnoid hemorrhage and status epilepticus. TCM has only been reported infrequently in association with traumatic brain injury (TBI). Herein we present a patient who developed TCM 3 days after hospital admission with severe TBI.

**Case Presentation:** A 30-year-old male presented to the hospital with an acute subdural hematoma, anisocoria, declining consciousness and CT evidence of uncal herniation after being found down in a hotel room. The patient was taken emergently to the operating room for decompressive hemicraniectomy and hematoma evacuation. On the post-trauma day (PTD) 3, the patient developed acute dyspnea with increased oxygen requirements that improved with diuresis. On PTD 4, nursing staff noted T waive inversions (TWI) on the bedside monitor prompting an electrocardiogram (ECG) that showed a prolonged QTc interval and worsening TWI in leads I, II, aVL, and V2-6. Troponin I level was mildly elevated at 0.63ng/mL. Transthoracic echocardiography (TTE) was subsequently performed and showed a low ejection fraction (EF 26%) with apical hypokinesis and basal hyperkinesis, consistent with TCM. A diagnosis of TCM was confirmed by Cardiology consultation and he was started on a beta-blocker and an ACE inhibitor. Follow-up TTE on PTD 20 showed a normal left ventricular EF.

**Conclusion:** While rarely reported in patients with TBI, TCM developed in an otherwise healthy young male following severe TBI necessitating decompressive hemicraniectomy. TTE should be considered in patients with TBI who have cardio-pulmonary symptoms or unexplained ECG abnormalities.

## Introduction

Takotsubo cardiomyopathy (TCM) or “stress” cardiomyopathy is characterized by transient left ventricular dysfunction with apical hypokinesis and compensatory basal hyperkinesis on cardiac imaging. TCM typically occurs 1–5 days after the inciting stressor. It predominantly affects elderly females who have suffered from significant physical or emotional stress (1). It has been reported in a variety of acute neurological conditions most prominently in patients with aneurysmal subarachnoid hemorrhage and status epilepticus (2). The association between traumatic brain injury (TBI) and TCM has only been reported infrequently in the literature. Herein we report a patient with severe TBI who developed evidence of TCM 3 days after the injury. This case report was prepared following the CARE Guidelines (3).

## Case Description

**Figure 1:**
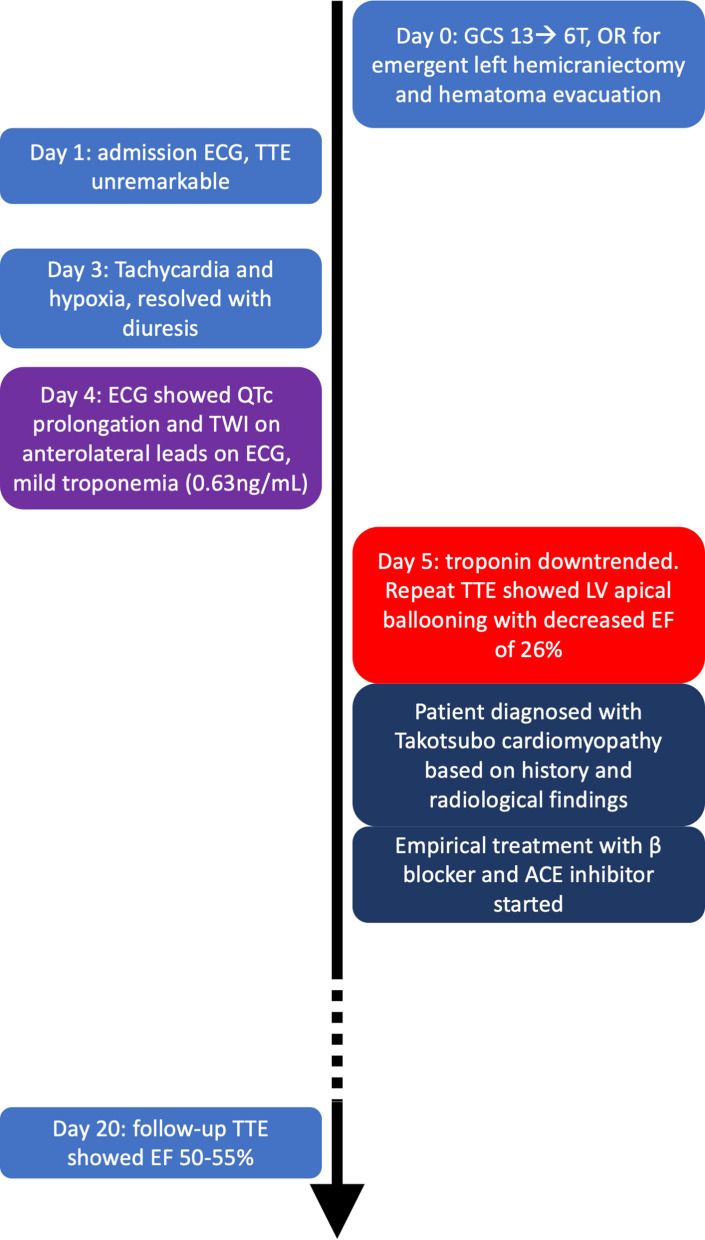
Timeline of presentation and outcome in this case.

**Figure 2:**
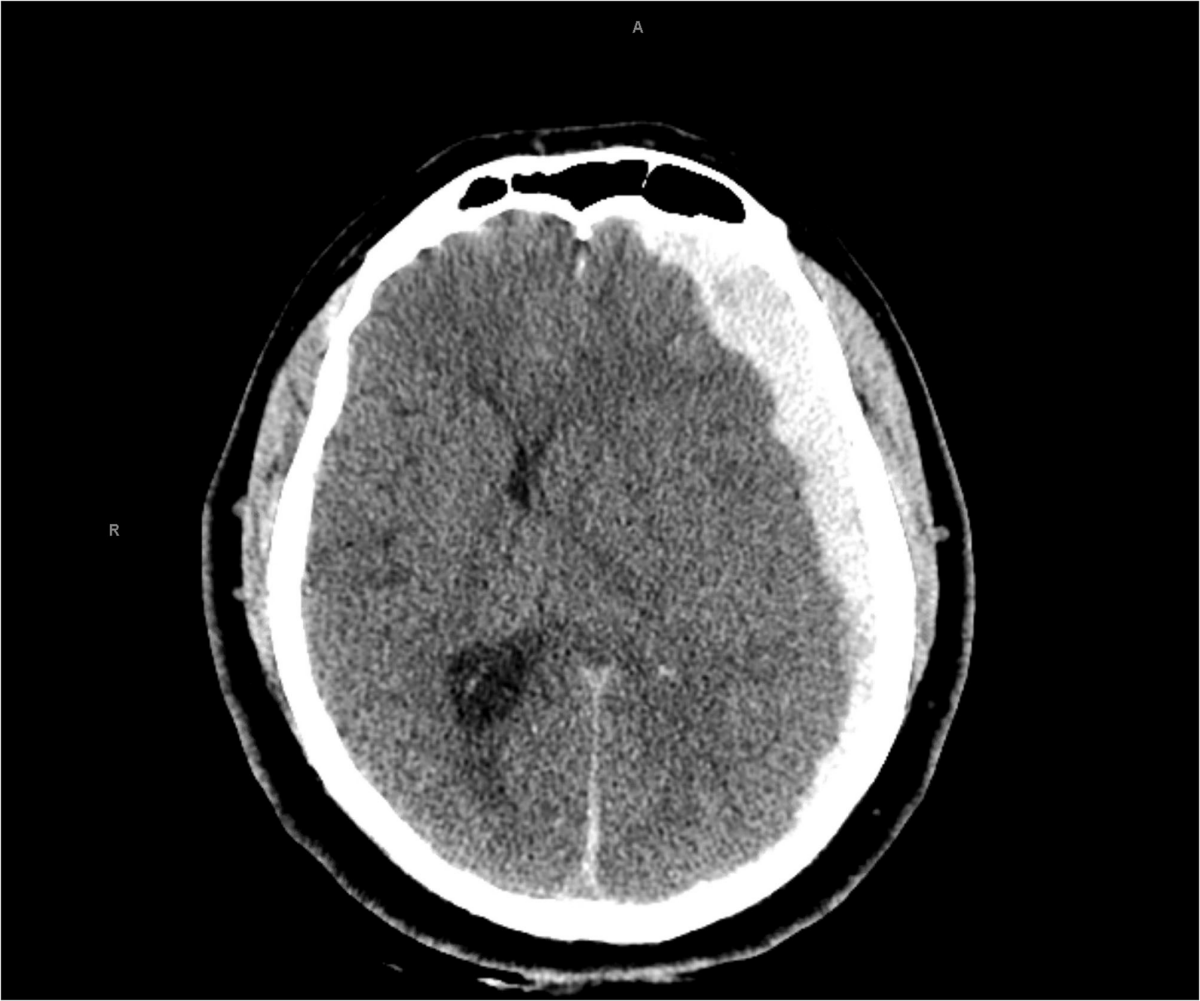
Computed tomography head showed an acute left subdural hemorrhage with significant left-to-right midline shift.

**Figure 3:**
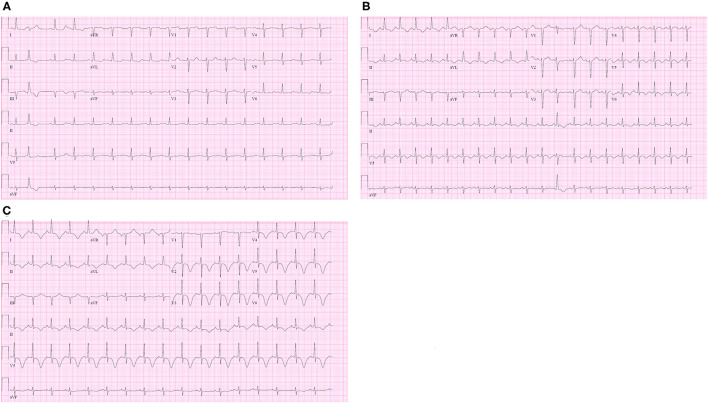
**(A)** Admission electrocardiogram (ECG) showed normal QTc interval and non-specific T wave changes; **(B)** ECG on the post-trauma day (PTD) 3 revealed new-onset QTc prolongation and T wave inversion on the lateral leads (aVL, V5, and V6); **(C)** ECG on PTD 4 revealed worsening TWI on leads I, II, aVL and V2-6.

A 30-year-old man with history of alcohol use was found unresponsive with unknown down time in his hotel room by the hotel staff. While his initial Glasgow Coma Score (GCS) was 13 at the scene, his GCS rapidly declined after arriving in the trauma bay and was emergently intubated for airway protection. Neurological examination revealed a fixed and dilated pupil on the left side with GCS of 6T after a brief sedation interruption (Figure 1). Secondary trauma survey showed ecchymosis on the left chest, shoulder and knee. Computed tomography (CT) scan of the head showed a 19 mm left convexity acute subdural hematoma with 14 mm of midline shift as well as uncal herniation (Figure 2). CT scan of the chest showed small pneumomediastinum and mild left-sided pneumothorax. His toxicology screen was only positive for ethanol with a level of 45 mg/dL. TBI was assumed given the constellation of evidence including CT scan findings, external trauma to the left side of body and positive ethanol level on initial toxicology screen. The patient was taken emergently to the operating room for decompressive hemicraniectomy and hematoma evacuation. Intra-operative bronchoscopy and esophagogastroduodenoscopy did not reveal additional injuries to the bronchial trees or esophagus. Postoperatively, the patient was admitted to neurotrauma ICU for close monitoring and his GCS improved to 10T. On the post-trauma day (PTD) 1, the patient had a routine admission electrocardiogram (ECG) that showed non-specific T wave changes (Figure 3A) with a normal ejection fraction (EF) on transthoracic echocardiography (TTE). He was successfully extubated on PTD 2 with an improved GCS of 13 (E3V4M6).

On the early morning of PTD 3, the patient developed tachycardia and acute hypoxia. Chest X-ray demonstrated mild pulmonary edema bilaterally. ECG was obtained which revealed new QTc prolongation and T wave inversion (TWI) on the lateral leads (aVL, V5, and V6). (Figure 3B) His oxygen supplementation was escalated to high flow nasal canula at 45 liters per minute. Hypoxia was attributed to pulmonary edema secondary to aggressive fluid resuscitation following the injury. Subjectively, the patient denied any chest discomfort and thus the ECG changes were deemed to be non-specific. His respiratory status rapidly improved with diuresis, and supplemental oxygen was weaned off on the same day.

On the night of PTD4, the bedside nurse noted obvious TWI on the bedside monitor. That prompted a repeat ECG demonstrating a persistently prolonged QTc interval and worsening TWI on leads I, II, aVL, and V2-6. (Figure 3C) The patient continued to deny any chest discomfort or shortness of breath. Troponin I was obtained given these new ECG changes and returned mildly elevated at 0.63 ng/mL (normal range 0–0.1 ng/mL).

By the next morning, repeat ECG was unchanged while troponin I downtrended to 0.54 ng/mL and then to 0.36 ng/mL. Repeat TTE was performed and demonstrated left ventricular apical ballooning with an estimated EF of 26% (Supplementary Video 1). Clinically, the patient had no symptoms of heart failure. Cardiology consultation recommended the initiation of lisinopril and metoprolol for the management of TCM. For the remainder of his hospitalization, he was monitored in the telemetry unit until discharge to a rehabilitation facility. Follow-up TTE on PTD 20 revealed improvement in EF to 50–55%.

## Discussion

Takotsubo cardiomyopathy is a form of stress-induced cardiomyopathy that characteristically ensues a few days after an inciting physical or emotional stressor event. Although the precise incidence of TCM remains unknown, the literature suggests an incidence of between 15 and 30 cases per 100,000 per year in the United States (4). In a series of 324 patients diagnosed with TCM, mean age was 68 years with more than 80% of patients being older than 50 years old (1). Females are reported to be 10.8 times more susceptible to TCM than males (5).

Acute neurological disorders, including aneurysmal subarachnoid hemorrhage and status epilepticus are known to be strongly associated with TCM (2). One retrospective study in patients with isolated TBI have reported myocardial dysfunction in 22% of patients: 12% of them had documented decreased left ventricular EF and 17.5% of patients had regional wall motion abnormality (6). Another pilot cohort study reported that 20% of isolated TBI patients developed myocardial injury, however it was defined by elevated levels of high-sensitivity troponin rather than echocardiographic changes (7). In contrast to these reports, a small prospective study in 49 patients with moderate to severe TBI with a median age of 34 years showed no significant impairment in cardiac function early after injury (8). TCM has only been infrequently reported in patients with TBI. In a case report with review of existing literature, only 17 cases of TCM with TBI had been reported in the literature as of 2017 (9). Notwithstanding these case reports, a large cross-sectional study of hospital admissions from the National Inpatient Sample concluded that TBI was negatively associated with development of TCM (2). However, that TBI is associated with cardiac dysfunction was shown in a recent prospective cohort study of 100 patients with moderate to severe TBI where there was evidence of impaired left ventricular function on speckle-tracking echocardiography in approximately 10% of patients (10). Speckle-tracking echocardiography is a more sensitive method than traditional TTE for determining left ventricular function as measured by changes in global longitudinal strain. While the risk of developing TCM in the TBI population is uncertain, there may be a spectrum of cardiac dysfunction in this population with TCM being on the extreme end of this spectrum.

The brain-heart interaction has been proposed a long time ago, however, it is unclear as to why emotional and/or physical stress leads to cardiac dysfunction in some patients but not others. Twenty patients during the acute phase of TCM underwent functional MRI with the goal to identify which brain structures are associated with development of TCM (11). Compared to normal controls, patients with TCM had homogenously increased blood flow to hippocampus, amygdala, precentral gyri and basal ganglia. The complex neuronal network reactions to stress are mediated by noradrenergic neurons and stress-related neurotransmitters. Supraphysiologic levels of circulating stress hormones may emulate indirect cardiac toxicity via either multi-vessel epicardial coronary spasm or microvascular dysfunction, which is similar to that observed in patients with pheochromocytoma (12, 13). The other possibility is that the overproduction of these stress-related hormones from presynaptic nerve endings to the heart poses a direct toxic effect leading to myocardial necrosis (14). Catecholamines bind to myocardial receptors and the ensuing downstream effects may lead to intracellular calcium overload, depressed myocardial contractility and eventually decline in cardiac function (15). Whether increased circulating catecholamine levels are causal or simply an epiphenomenon secondary to myocardial stress and decreased cardiac output remains uncertain.

The essential elements in establishing a diagnosis of TCM is the demonstration of new-onset transient LV regional wall motion abnormalities that extend beyond a single coronary artery territory on cardiac imaging (16). An emotional or physical trigger may or may not be identified in clinical practice. Other commonly performed tests include ECG and cardiac biomarkers. ECG may demonstrate new abnormalities such as ST-segment elevation, ST-segment depression, TWI, and QTc prolongation. Cardiac biomarkers (troponin and creatine kinase) are moderately elevated in most cases; significant elevation of brain natriuretic peptide is also common (16). Most importantly, irreversible coronary artery occlusion must be ruled out prior to establishing the final diagnosis. Thus, many patients with TCM undergo coronary angiography to rule out underlying coronary artery disease as the cause for cardiac dysfunction. In our patient, coronary angiography was not pursued before establishing the final diagnosis given that he was young, did not have any obvious risk factors for coronary artery disease and had a clear inciting stressor event prior to the development of symptoms. The timing of the onset of manifestations including dyspnea and pulmonary edema combined with characteristic findings on ECG and TTE were sufficient to establish the diagnosis. Finally, his troponin I level was only mildly elevated. While coronary angiography with the potential for PCI is commonly performed to exclude acute coronary disease in TCM, the totality of the clinical picture combined with absolute contraindications to anticoagulation or antiplatelet therapy militated against pursuing the possibility of coronary artery disease. Given all the clinical data, a presumptive diagnosis of TCM was established.

Patients with TCM are generally considered to have a good prognosis. In a prospective registry of 100 patients with TCM, 96 had recovery of cardiac function, although it might take a few weeks to months (17). Contemporary data have also shown that TCM may have major complications, ranging from acute heart failure and cardiogenic shock to dysrhythmias and thromboembolism (18). Fortunately, our patient did not present with any major complications of TCM with the exception of acute pulmonary edema. His cardiac function also recovered after 3 weeks.

## Conclusion

This case emphasizes that TCM does occur in the setting of acute TBI. Although uncommonly reported, cardiac dysfunction may be more frequent than previously thought especially when sensitive methods for detecting left ventricular dysfunction are used. TCM should be considered as a recognized complication of TBI with the potential complications including manifestations related to impaired left ventricular function.

## Data Availability Statement

The original contributions presented in the study are included in the article/Supplementary Material, further inquiries can be directed to the corresponding author/s.

## Ethics Statement

Written informed consent was obtained from the patient for the publication of any potentially identifiable images or data included in this article.

## Author Contributions

FW: study concept and design, data acquisition, first draft, and literature review. JD: study concept and design, literature review, and revision of the manuscript for intellectual content. All authors contributed to the article and approved the submitted version.

## Funding

Department of Critical Care Medicine at University of Pittsburgh Medical center will support publication fee.

## Conflict of Interest

The authors declare that the research was conducted in the absence of any commercial or financial relationships that could be construed as a potential conflict of interest.

## Publisher's Note

All claims expressed in this article are solely those of the authors and do not necessarily represent those of their affiliated organizations, or those of the publisher, the editors and the reviewers. Any product that may be evaluated in this article, or claim that may be made by its manufacturer, is not guaranteed or endorsed by the publisher.

## Supplementary Material

Supplementary Video 1 TTE on the apical view demonstrating characteristic apical hypokinesis with compensatory basal hyperkinesis.
